# Online health information–seeking behaviours for low back pain in the United Kingdom: analysis of data from Google trends and the Global Burden of Disease Study, 2004–2019

**DOI:** 10.1093/inthealth/ihae020

**Published:** 2024-02-23

**Authors:** Harpal Patel, Thomas A Shepherd

**Affiliations:** School of Medicine, Keele University, Staffordshire ST5 5BG, UK; School of Medicine, Keele University, Staffordshire ST5 5BG, UK

**Keywords:** disability, Global Burden of Disease, Google Trends, infodemiology, low back pain, online health information seeking

## Abstract

**Background:**

Low back pain (LBP) is a leading cause of global disability. Timely health-seeking is crucial for early diagnosis and management of pathologies. Despite increases in internet usage, there is sparse literature regarding online health information–seeking behaviours (OHISBs) for LBP and how they correlate with the LBP disease burden in the UK.

**Methods:**

To examine OHISB trends, we conducted Prais–Winsten analyses on monthly search volume data from Google Trends in the UK between 1 January 2004 and 1 December 2019. Cross-correlation analyses assessed the relationship between annual LBP search volume and LBP morbidity and mortality data from the Global Burden of Disease study (2004–2019).

**Results:**

From 2004 to 2019, the trend in LBP search volume was curvilinear (β=1.27, t=5.00, p<0.001), with a slope change around the end of 2006. There was a negative linear trend (β=−0.25, t_35_=−1.52, p<0.14) from 2004 to 2006 and a positive linear trend (β=0.67, t_108_=9.17, p<0.001) from 2007 to 2019. Cross-correlations revealed positive associations between search volume and disease burden indicators for LBP such as prevalence and incidence at lags 4 and 5.

**Conclusions:**

A rising trend in OHISBs for LBP was noted between 2004 and 2019. This trend positively correlates with incidence, prevalence and burden measures. These findings emphasise the importance of high-quality online resources to increase awareness around LBP, facilitating early diagnosis and management.

## Introduction

Low back pain (LBP) is a common and debilitating symptom that can manifest in people of all ages as a result of various aetiologies.^1,2^ It is defined as pain in the area between the lower boundary of the 12th rib and the lower gluteal folds, with possible associated radiation to the lower limbs.^3^ It is currently the leading cause of disability globally, measured by years lived with disability (YLD).^3^ In most cases, a definite nociceptive source cannot be identified and therefore it is classed as ‘non-specific’.^4^ In the current ageing population, the prevalence of LBP continues to increase rapidly. As of 2020, it was estimated to affect around 619 million people globally and is projected to reach 843 million by 2050, driven by population growth.^5^ A study on 15 272 adults in the UK found the 1-month period prevalence of LBP to be 28.5%, and increasing with age, with the largest prevalence among those ages 41–50 y.^6^ In the UK, LBP is responsible for huge costs, both directly due to medical care required for patients, and indirectly due to lost productivity in the workplace or household. The National Health Service (NHS) estimates healthcare costs for patients with LBP to be around £1.6 billion.^7^ It is estimated that employees with back pain take an average of 14–24 sick days per year.^8^ Work absences due to LBP, termed ‘absenteeism’, and decreased productivity while working with back pain, termed ‘presenteeism’, collectively account for an indirect cost to the UK economy of approximately £5–10.7 billion.^7^ LBP is an important symptom to resolve quickly due to its link with multiple comorbidities such as poor mental health, including depression, anxiety and insomnia.^9^ Additionally, severe chronic pain is associated with a significant increase in all-cause mortality, independent of other confounding factors such as socio-economic status.^10^

Despite the huge prevalence and debilitating nature of LBP, there remains a large gap in awareness and understanding among the public. Barriers in seeking healthcare for LBP, such as knowing when to seek help, what the red flags are and the physical barriers (e.g. mobility issues), prevent the prompt and accurate diagnosis of LBP^11^ and subsequent timely access to a range of management options. With the increase in global internet use in the past 2 decades, searching for health information online has increased.^12,13^ In 2021, the internet penetration for the UK was at 96.7%.^14^ A study found that of 155 sampled participants, 65.8% had searched for their minor pain symptoms on the internet, with most of these patients sharing their findings with their doctors.^15^ The internet can provide a huge dataset to monitor health information–seeking behaviours in real time. Individuals with symptoms of LBP may engage in online health information–seeking behaviours (OHISBs), however, it is unclear if these behaviours correlate with diagnoses and management.

A popular way of obtaining data on OHISBs is through Google searches, as it is the world's most utilised search engine. Google Trends (GT) is a free, publicly accessible tool that provides data on which Google searches are ‘trending’ at any given time in a particular geographical area. It contains search data from 2004 to the present, and the data can be a proxy marker for disease awareness and possibly disease incidence and prevalence (i.e. if an individual searches for symptoms of LBP, they are likely to have it). GT data has been utilised in the past to understand OHISBs and search patterns for various diseases, including COVID-19, Mpox, chronic obstructive pulmonary disease (COPD), and the effectiveness of public health days on increasing awareness about public health topics.^16–19^

In this study, we aim to examine data on LBP gathered from GT to determine trends in OHISBs over time and if they are correlated with data points from the Global Burden of Disease (GBD) study (2019) such as prevalence, incidence and disability.

## Methods

### Search data from GT

GT data were obtained from a sample of all Google searches for a given time period. These data were categorised, linked to specific topics and anonymized. In order to assess relative popularity accurately, GT normalises every data point by dividing it by the total searches within the geographical area at a particular point in time. This normalisation prevents areas with the highest search volume (i.e. highest population) from consistently ranking at the top. The resulting numbers are scaled on a range of 0–100 to designate the ‘popularity’ of a topic, taking into account a topic's proportion in relation to all searches across various topics.^20^ Only the data point from the initial Google search is included within the GT algorithms; any subsequent browsing activity is not recorded. To increase the reproducibility of the findings, methods are detailed following the recommended reporting guidelines.^21^

### GBD 2019

The GBD 2019 study examined 369 diseases and injuries in 204 countries between 1990 and 2019.^3^ These studies are conducted annually by the Institute for Health Metrics and Evaluation and provide large-scale, free, publicly accessible data regarding LBP. They cover parameters such as incidence, prevalence, disability-adjusted life years (DALYs), years lived with disability (YLD) and mortality. Incidence reflects the number of new cases observed for LBP at a particular time, while prevalence indicates the total proportion of the population experiencing this condition. YLD quantifies the amount of healthy life lost due to poor health; 1 YLD signifies the loss of 1 y of healthy life. DALYs encompass both YLD and years of life lost (YLL) due to premature mortality. A single DALY represents the equivalent of 1 y of healthy life lost due to either poor health or premature death.^22^ The GBD 2019 study draws from diverse data sources, including published literature, hospital and clinical data, surveillance and survey data and medical records from inpatient and outpatient settings.^3^

### Search input

Upon searching ‘low back pain’, there are multiple types of search entry types, including ‘low back pain’ as a search term and as a disorder. For this study, ‘low back pain’ as a search term was selected, as it encompasses multiple similar search terms with the same meaning, including ‘back pain’, ‘pain low back’ and ‘back pain low’, and it also includes translations of ‘low back pain’ in other languages.

### Search date

Monthly data for LBP was obtained from GT for the UK between January 2004 and December 2019. The rationale for choosing this time period was that the first GT data were available in January 2004, while December 2019 is the last data point available for the GBD study. Data were accessed and downloaded on 22 June 2023 and all query categories were used. The monthly relative search volume (RSV) data, ranging from 0 to 100, were converted to an annual average over 12 months (using the mean of the 12 months) and compared with the parameters from the GBD study side by side for each year from 2004 to 2019.

### Data analysis

The data were analysed using SPSS 29.0 statistics software (IBM, Armonk, NY, USA). Analysis was first conducted on the monthly GT RSV data to deduce if autocorrelation existed within the monthly data. To determine autocorrelation, a Prais–Winsten regression was carried out, and a Dubin–Watson statistic for autocorrelation was calculated. Monthly GT data were converted into a yearly mean for the period 2004–2019, after which a cross-correlation analysis was done using the incidence, prevalence, YLD and DALYs data from the GBD 2019 study for the same period.

## Results

### Trends in monthly search volume for LBP from January 2004 to December 2019

**Figure 1.**
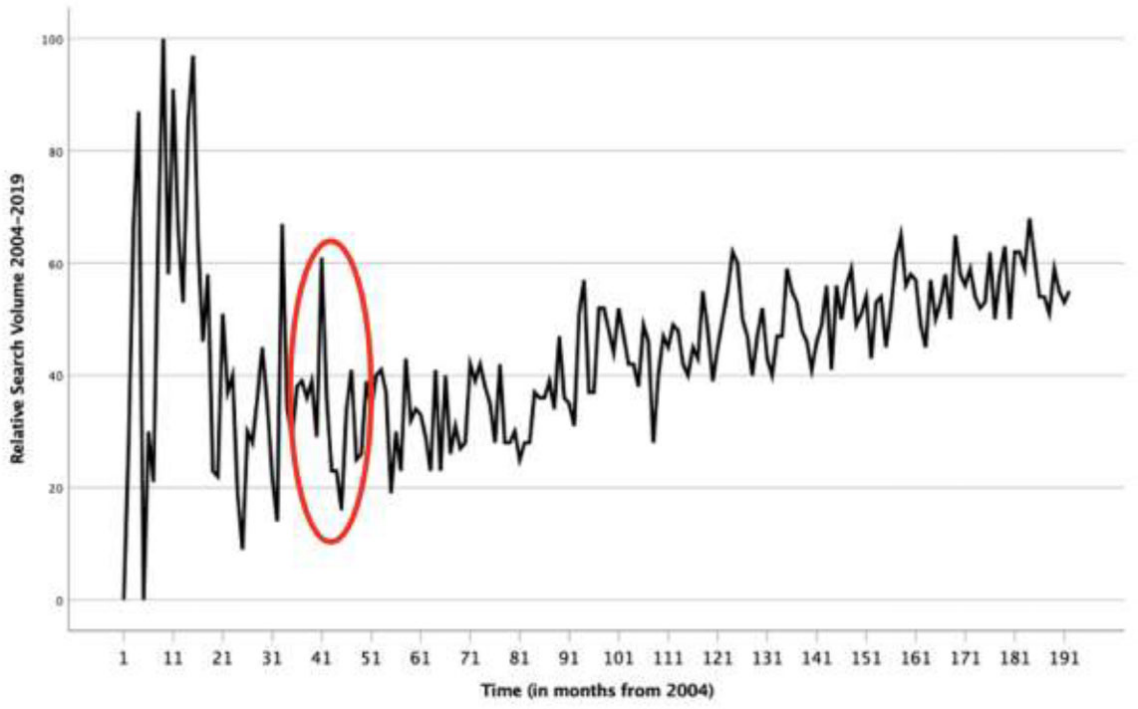
GT RSV for LBP from 2004 to 2019. The apparent slope change in RSV is indicated by the red circle.

The trends in monthly search volume for LBP between January 2004 and December 2019 are shown in Figure 1.

An overall positive correlation is seen with RSV over time. The Durbin–Watson statistic^23^ was 1.20. The Prais–Winsten regression of the whole study period, January 2004–December 2019, resulted in an adjusted R^2^ value of 0.23 (standard error [SE] 13.39; unadjusted R^2^ = 0.24). The autocorrelation coefficient was 0.37 (SE 0.68). Time demonstrated a quadratic effect on search volume (β = 1.27, t = 5.00, p < 0.001), as shown in Table 1. A quadratic effect suggests that one slope change occurred over the time period.

**Table 1.** Prais–Winsten regression examining the effect of time on monthly search volume for LBP (2004–2019)

Predictors	Beta (standardised coefficients)	t (df=191)	p-Value
Time	−0.86	−3.38	<0.01
Time^2^	1.27	5.00	<0.001

df: degrees of freedom.

In Figure 1, the slope appears to change around early to mid 2007, circled in red. To examine the trend before and after the apparent slope change, Prais–Winsten regressions were conducted for the two periods: 2004–2006 (36 months) and 2007–2019 (156 months). Lastly, an analysis was carried out for the period 2011–2019 due to a change in the GT algorithm for calculating RSV, which took effect on 1 January 2011. These analyses are presented in Table 2.

**Table 2.** Prais–Winsten regression analyses examining the effect of time on monthly search volume for LBP in 2004–2006, 2007–2019 and 2011–2019

Predictor: time	Beta (standardised coefficients)	t	p-Value
2004–2006	−0.25	−1.52 (df=35)	<0.14
2007–2019	0.78	15.55 (df=155)	<0.001
2011–2019	0.67	9.17 (df=107)	<0.001

df: degrees of freedom.

From 2004 to 2006, the Durbin–Watson statistic was 1.32 and the autocorrelation coefficient was 0.30 (SE 0.17). For the overall model, the adjusted R^2^ was 0.071 (SE 25.58) (unadjusted R^2^ = 0.12). Time demonstrated a negative linear effect on search volume (β = −0.25, t_35_ = −1.52, p<0.14), suggesting a reduction in monthly search volume during this period.

From 2007 to 2019, the Durbin–Watson statistic was 1.56 and the autocorrelation coefficient was 0.21 (SE 0.08). For the overall model, the adjusted R^2^ was 0.61 (SE 7.11) (unadjusted R^2^ = 0.61). Time demonstrated a positive linear effect on search volume (β = 0.78, t_155_ = 15.55, p<0.001), suggesting that monthly search volume exhibited a positive linear trend from 2007 to 2019.

From 2011 to 2019, the Durbin–Watson statistic was 1.94 and the autocorrelation coefficient was 0.20 (SE 0.10). For the overall model, the adjusted R^2^ was 0.34 (SE 5.96) (unadjusted R^2^ = 0.36). Time demonstrated a positive linear effect on search volume (β = 0.67, t_108_ = 9.17, p < 0.001), suggesting a positive linear trend consistent with the trend from 2007 to 2019, despite the changes to GT on 1 January 2011.

### Cross-correlation between annual search volume and disease burden indicators for LBP (2004–2019)

A mean was taken of the monthly RSV data from GT to give an annual average for RSV, allowing a comparison with the annual data from the GBD study. Table 3 presents cross-correlations between annual RSV and disease burden indicators at lags −1, 0, 1, 2, 3, 4 and 5.

**Table 3.** Cross-correlation analysis of annual LBP search volume and LBP disease burden indicators (2004–2019)

Predictor	Lag −1	Lag 0	Lag 1	Lag 2	Lag 3	Lag 4	Lag 5
Incidence	0.001	−0.176	−0.356	−0.341	−0.217	0.018	0.380
Prevalence	−0.127	−0.363	−0.565*	−0.589*	−0.454	−0.186	0.141
YLD	−0.133	−0.367	−0.567*	−0.589*	−0.451	−0.185	0.141
DALYs	−0.133	−0.367	−0.567*	−0.589*	−0.451	−0.185	0.141

*Exceeds 95% confidence interval.

Cross-correlation analysis revealed that there was a negative correlation between RSV and incidence, prevalence and YLD/DALYs data at lag 0. There was a positive correlation between RSV and incidence at lag 4, suggesting a 4-y delay between online searching and a diagnosis of back pain. A positive correlation between RSV and prevalence, and RSV and YLD/DALYs became apparent at lag 5, suggesting a 5-y delay.

## Discussion

This study examined trends in OHISBs for LBP in the UK between 2004 and 2019 and explored the relationship between those trends and several GBD indicators, including incidence, prevalence, YLD and DALYs.

### Principal findings

Findings show that the OHISBs for LBP have increased over time between January 2004 and December 2019. In a search of news and events from 2004 to 2019, there were no significant events such as LBP awareness campaigns that explain this positive trend. The trend in RSV for LBP was curvilinear, with a slope change at the end of 2006. Further analyses revealed a downward trend in RSV from 2004 to 2006 and an upward trend from 2007 to 2019. The downward trend from 2004 to 2006 was not expected and could have been due to a multitude of factors. One possible explanation is ‘noise’ in the data caused by the GT algorithm still being in its infancy at the time, given the fact that GT only began as a project in 2004. Their data collection and analysis methods were improved in later updates, in January 2011 and January 2016, which could explain the more consistent trend from 2007 onwards.^24^ Another potential explanation is that between 2004 and 2006, limited use of personal computers and the internet may have resulted in infrequent searches for LBP, possibly contributing to fluctuating trend results at both extremes. Access to the internet was significantly better from 2007 onwards,^25^ likely due to the increase in the use of computers and smartphones, revolutionised by the Apple iPhone, which was introduced that same year.^26^

Results revealed a positive correlation between search volume and GBD indicators, such as incidence and prevalence. This was evident on the fourth and fifth lag, suggesting there is a 4- to 5-y delay between searching for LBP online and the diagnosis made by a healthcare professional. One potential explanation for this delay could be that patients could have initially searched for symptoms online and were able to manage their back pain using over-the-counter analgesics. However, when symptoms became more severe or became more intrusive to their activities of daily living, patients sought professional advice and received a diagnosis. This could particularly be the case for younger individuals, who may put off seeing their doctor due to work or other commitments, explained further in the limitations. Another potential reason for this delay is the long waiting times to be seen by specialists and patients often requiring multiple visits to the doctor before a definitive diagnosis is reached.^27^ Indicators like YLD and DALYs showed a 5-y delay from symptom searching, implying that it took 5 y after the initial OHISB for years of life to start being lost due to disability. This delay can also be explained by the worsening of symptoms and impact on activities of daily living of patients over time.

This study demonstrates that an increasing number of patients seek information about their LBP symptoms online. The positive correlation of search volume with GBD indicators implies that increases in the incidence and prevalence of LBP is reflected in OHISBs. Patients with LBP, or their caregivers, may be searching online for the cause of their pain or to find treatments to help manage the pain. Additionally, health information–seeking behaviours could be a key strategy for patients to cope with their back pain.^28^ Although most people rely on their doctors as their primary source of health information, sometimes they are unsatisfied with the lack of medical explanations and lack of adequate solutions for their pain and resort to seeking information online.^29^ Some patients also opt for alternative medicine such as acupuncture, massage and spinal manipulation.^30^ Another important factor to consider is timeliness; patients may seek information from online sources because it is quicker. This is particularly important in light of the current crisis where NHS waiting times are at an all-time high for accident and emergency and outpatient appointments, e.g. orthopaedics and rheumatology.^31^ There are also several patient-related barriers to a timely diagnosis when presenting with LBP. For instance, patients may have insufficient knowledge about back pain to know when to seek help. They may be unaware of the red flag signs associated with LBP, such as weight loss and incontinence, which could lead to missed diagnoses of serious lower back pathologies. Another barrier is that individuals do not prioritise seeking advice from a healthcare professional about their LBP, whether it be due to work commitments, personal beliefs or that symptoms are not yet severe enough to warrant seeking help. This can make it difficult for an early diagnosis to be reached, which can impede early treatment, such as physiotherapy. A randomised clinical trial has demonstrated that early physiotherapy provides a significant improvement in disability in individuals diagnosed with LBP compared with usual care alone.^32^

### Recommendations

Based on the findings of this study, emphasis should be placed on increasing awareness regarding LBP so that patients can receive an early diagnosis and early treatment, before symptoms become severe or hinder their activities of daily living. One way this could be done is through online health campaigns focusing on LBP awareness, which could aim to explain the causes of LBP, red flag symptoms to be aware of and when to seek advice from a healthcare professional. This could be in the form of a website or app; however, it is crucial to ensure that it is a user-friendly interface, as patients report that as a key barrier to accessing health information online.^30^

### Strengths and limitations

A key strength to this study is that it is one of the first studies examining OHISBs for LBP through GT data in the UK. Furthermore, this study compares the correlation between GT data and GBD indicators to understand if OHISBs can predict the incidence and prevalence of LBP, which has not been done previously. The results of this study are supported by other evidence from research examining OHISBs for COPD using GT data,^16^ suggesting a general increase in OHISBs for various medical conditions over time as internet use increases.

A few limitations of this study should be considered. First, the exact data collection and analysis methods for GT data are unknown. Second, as GT data are reliant on the internet, it is difficult to understand OHISBs in certain groups of people who do not or cannot access the internet, such as the elderly.^31^ One study found that younger patients and females were more likely to use the internet to search for health information.^32^ Younger patients, who often have work commitments, may be more likely to tolerate back pain symptoms for longer and may try other remedies such as over-the-counter analgesics initially, resulting in a delayed consultation with their doctor. This could explain why a large lag exists between symptom searching and case incidence and prevalence. Another limitation is that GT has made several improvements to its data collection and analysis over the years, making it difficult to understand RSV over different time periods (e.g. before and after an improvement). However, our analysis of the data in different years (2004–2006 and 2007–2019) has helped mitigate any differences caused by this. Finally, it is important to note that OHISBs occur through various other platforms apart from Google searches, such as through other search engines and social media. This is an area that requires further research, as the prospect of delivering health information through social media is currently an area of great interest.

## Conclusions

Analysis of Google search data revealed an increasing trend in OHISBs regarding LBP in the UK between 2004 and 2019, which positively correlates with GBD incidence and prevalence indicators over the same period. These findings suggest an increasing number of patients, relatives and caregivers are accessing health information regarding LBP online and that internet searching patterns may be linked with a diagnosis by a healthcare professional. This emphasises the need for accurate and high-quality informational material regarding LBP to be available on the internet in a user-friendly manner for people of all ages. This can contribute towards early diagnosis and prompt patient-centred treatment in order to achieve better health outcomes for patients with LBP.

## Data Availability

Data are available upon reasonable request to the corresponding author.
